# Gonadotropin-Releasing Hormone (GnRH) Agonist Protocol Improves Pregnancy Outcomes During In Vitro Fertilization (IVF) and Intracytoplasmic Sperm Injection (ICSI) Treatment in Young Infertile Women: A Retrospective Study

**DOI:** 10.7759/cureus.61554

**Published:** 2024-06-02

**Authors:** Aamir Mahmood, Li Tan

**Affiliations:** 1 Reproductive Medicine, The Second Affiliated Hospital of Zhengzhou University, Zhengzhou, CHN

**Keywords:** in vitro fertilization (ivf), female infertility, gonadotropin-releasing hormone (gnrh) antagonist, gonadotropin-releasing hormone (gnrh) agonist, intracytoplasmic sperm injection (icsi)

## Abstract

Objective

The objective of this study was to determine if gonadotropin-releasing hormone agonist (GnRH-a) or gonadotropin-releasing hormone antagonist (GnRH-ant) protocols during in vitro fertilization (IVF) or intracytoplasmic sperm injection (ICSI) treatment in young infertile women improve their pregnancy outcomes.

Methodology

We retrospectively reviewed the records of 876 young infertile women aged 20-35 years who underwent fresh embryo transfer in IVF/ICSI cycles. The data were collected from their initial visits to the reproductive medicine center of the Second Affiliated Hospital of Zhengzhou University between January 2019 and December 2022. We divided them into two groups according to the controlled ovarian hyperstimulation (COH) protocols: GnRH-a (*n* = 580) and GnRH-ant (*n* = 296). The primary outcome assessed in this study was the live birth rate. The secondary observation indicators included the total dose and duration of gonadotropin (Gn), total embryo transfer, day three (D3) embryo transfer, total two pro-nuclei (2PN) cleavage count, number of fertilizations, and implantation rate.

Results

The live birth rate had no clinical significance (*P* > 0.05). The total dose and duration of Gn stimulation in the GnRH-ant group were lower than in the GnRH-a group (*P *< 0.05). The total embryo transfer, D3 embryo transfer, total cleavage count, total 2PN cleavage count, number of fertilizations, transfer, and mature oocytes in metaphase II (MII) of D3 embryos in the GnRH-a group were higher than those in the GnRH-ant group (*P *< 0.05). The clinical pregnancy rate and implantation rate of the GnRH-a group were higher than those of the control group.

Conclusions

The total embryo transfer, D3 embryo transfer, total cleavage count, total 2PN cleavage count, number of fertilizations, transfer and MII of D3 embryos, clinical pregnancy, and implantation rates were significantly higher in the GnRH-a protocol group. The total dosage of Gn and duration of Gn stimulation were lower in the GnRH-ant group than in the GnRH-a group. These findings provide the basis for the selection of the COH protocol in normal Chinese ovarian response patients undergoing IVF/ICSI.

## Introduction

Due to the rapid advancement of assisted reproductive technology (ART), physicians must create effective and secure ovulation induction protocols for their patients. Theoretically, the gonadotropin-releasing hormone antagonist (GnRH-ant) protocol offers benefits like easy administration, a brief treatment duration, and a low ovarian hyperstimulation syndrome (OHSS) risk. Currently, the GnRH-ant protocol is predominantly administered to high or poor ovarian responders. There has been a debate on the suitability of the GnRH-ant protocol for normal ovarian responders [1-4]. There is a lack of consistency in the reports of clinical outcomes for the antagonist regimen following fresh embryo transfer. A meta-analysis of five randomized controlled trials in normal ovarian responders found that the live birth rate was notably lower with the GnRH-ant protocol compared to the GnRH agonist (GnRH-a) protocol, indicating that the latter may be more appropriate for this group [5-6]. A retrospective study found that the GnRH-ant and the GnRH-a protocols had similar clinical and ongoing pregnancy rates. However, the antagonist protocol was more effective in easing patient discomfort and reducing the economic burden of such treatment [7-8].

This study aimed to offer additional clinical guidance for choosing ovulation induction protocols in normal ovarian responders by comparing clinical outcomes between the GnRH-ant and GnRH-a protocols. Even though a meta-analysis has revealed that antagonist regimens can decrease OHSS in individuals with normal ovarian reserve, they can still achieve similar clinical pregnancy and live birth rates as long agonist regimens. However, there are still numerous studies and systematic evaluations indicating that the embryo implantation rate and clinical pregnancy rate of fresh cycle antagonist regimens are lower than those of long agonist regimens [9-11], which are believed to be related to the reduced endometrial receptivity of antagonist regimens [12-14]. This study assessed whether agonist regimens could be more appropriate for individuals with normal responses in clinical settings. It retrospectively examined clinical characteristics and outcomes of in vitro fertilization (IVF) cycles in normal responders using antagonist and agonist regimens at our center, offering guidance for regimen selection and fresh cycle transplantation strategies for these patients.

## Materials and methods

Patient population

We conducted a retrospective analysis of normal ovarian responders who underwent IVF or intracytoplasmic sperm injection (ICSI) embryo transfer in the reproductive medicine center of the Second Affiliated Hospital of Zhengzhou University between January 2019 and December 2022. This research initially considered a total of 6,510 fresh transfer cycles. After applying screening criteria, 876 cycles were included in the analysis. Among these, there were 580 cycles in the GnRH-a group and 296 cycles in the GnRH-ant group. Data were taken from the hospital’s electronic patient record system. This study received approval from the hospital’s ethics committee, which waived the requirement for patient consent.

Inclusion and exclusion criteria

The inclusion criteria were (1) age between 20 and 35 years; (2) basal follicle-stimulating hormone (FSH) level below 10 IU/L; (3) anti-Mullerian hormone (AMH) level above 1.2 μg/L; (4) basal antral follicle count (AFC) of five or more; and (5) IVF/ICSI. The exclusion criteria were (1) patients with polycystic ovary syndrome (PCOS); (2) patients with ovarian insufficiency; (3) patients with abnormal uterine cavity impacting embryo implantation; and (4) patients needing genetic diagnosis before embryo implantation.

Research methods

GnRH-a Protocols

There are different GnRH-a protocols in practice. The long protocol is as follows: 0.1 mg started in the previous cycle’s follicular or luteal phase (cycle day 21) until human chorionic gonadotropin (hCG) administration. The short protocol usually begins on day 1 or 3 of stimulation and continues until hCG is administered. The ultrashort protocol is GnRH-a 0.1 mg administered on days 2-4 of stimulation. In clinical practice, long protocols last for four to six weeks, whereas short protocols last for two to three weeks until the day of hCG injection, though the duration may vary. Antral follicles are detected during a vaginal B-mode ultrasound. If all antral follicles have diameters below 10 mm, intramuscular injections of GnRH-a (Triptorelin, Ipsen Pharmaceutical Co., Ltd.) are administered, and the duration depends on which protocol is practiced. If after 28 days, the luteinizing hormone (LH) level is <5 mIU/mL, estradiol (E2) <50 pg/mL, endometrial thickness <5 mm, and follicular diameter <10 mm on ultrasound, 150-300 IU of Gn is initially administered, with further adjustments based on follicular growth and hormone levels. When the follicular diameter in two or more ovarian follicles reaches 18 mm, or 17 mm in three or more follicles, 250 μg of choriogonadotropin alfa (Ovidrel) is administered subcutaneously. Oocyte retrieval takes place approximately 34 to 36 hours later. If no abnormalities are present, one or two fresh embryos are transferred three days after the retrieval.

GnRH-ant Protocols

Currently, three GnRH-ant protocols are used: the fixed day 6 protocol, in which 0.25 mg GnRH-ant is given daily until hCG is administered; the single-dose protocol, in which 3 mg GnRH-ant is given on day 7 of stimulation; and the flexible-dose protocol, in which 0.25 mg GnRH-ant is administered when follicles reach >14 mm. During the menstrual cycle, a dose of 150-300 IU of Gn is administered, followed by adjusting the Gn dosage and providing an antagonist (Cetrorelix, Merck, Switzerland; or Ganirelix, MRK, The Netherlands) based on follicular growth until the day of hCG administration. When two or more ovarian follicles reach a diameter of 18 mm, or three or more reach 17 mm, a subcutaneous injection of 250 μg of Ovidrel or 0.2 mg of Diphereline along with 2,000 IU of hCG is administered. The oocyte retrieval procedure is conducted between 34 and 36 hours later. In the absence of any abnormal conditions, one or two fresh embryos are transferred three days after the oocyte retrieval.

Oocyte Retrieval

Following the injection of 20% FSH (Puregon, Australia) dosages, oocytes are retrieved via vaginal puncture with ultrasound guidance by skilled physicians. The puncture needle, attached to a negative pressure suction device, enters the ovary through the vaginal curvature and punctures follicles larger than 14 mm in size, starting proximally and moving distally. The follicular fluid is promptly transferred to the lab for the examination and grading of oocytes under a microscope.

The grading system for evaluating embryo quality is based on the grading system [15]. Day 3 (D3) embryos are categorized into four grades: Grade I has even blastomeres with regular shapes and intact zona pellucidae, with less than 10% cell debris. Grade II has slightly irregular blastomeres with granulation in the cytoplasm and 10-20% cell debris. Grade III has uneven blastomeres with prominent granulation and 20%-50% cell debris. Grade IV has severely uneven blastomeres with a lot of granulation and over 50% cell debris. Grade I and grade II embryos are considered to be of high quality.

In Vitro Fertilization/Intracytoplasmic Sperm Injection

IVF is conducted for four to six hours following egg retrieval. The process occurs in a culture dish, where sperm is activated by the proteins present. It then passes through the cumulus cells and corona radiata undergoes an acrosome reaction on the zona pellucida, dissolves it, and binds with the egg to complete gamete fusion. In contrast, ICSI is a technique used during IVF where a single sperm is injected directly into the egg for fertilization.

Outcome Measurement

Clinical outcomes included the clinical pregnancy and live birth rates. Clinical pregnancy is determined through ultrasonographic observation of at least one gestational sac or a fetal heartbeat within four to six weeks after embryo transfer. The live birth rate per cycle is calculated by dividing the number of pregnancies/live births achieved from the first to the current cycle by the initial number of patients, assuming no patient dropout. The implantation rate (IR) is calculated as the number of gestational sacs observed through vaginal ultrasound 3-5 weeks after transfer (ngestational) divided by the number of transferred embryos (ntransferred).

Embryo Transfer

A high-quality embryo is chosen and placed in the uterus 48 hours post-oocyte retrieval. The ultrasound guides the transfer tube with the embryo into the uterus. The embryo is placed 1 cm from the bottom of the uterus. After waiting for 30 seconds, the transfer tube is removed and the laboratory physician inspects it for any remaining embryo. If any residue is found, a second transfer is promptly carried out.

Luteal Support

After oocyte retrieval, both patient groups are administered a 60 mg/day intramuscular injection of progesterone (P) (Zhuhai Lizhu Pharmaceutical, Zhuhai, China). Estradiol valerate 2 mg (Bujiale, Guangzhou Xianling Pharmaceutical, Guangzhou, China) is given twice daily, beginning on the third day after embryo transfer. Serum hCG is tested on the 14th day after transfer to assess for biochemical pregnancy.

Statistical analysis

Statistical analysis was done using the IBM SPSS Statistics for Windows, version 26.0. (IBM Corp., Armonk, NY). The data were presented as mean ± standard deviation (x ± s) and analyzed using an independent samples t-test. Counting data were presented as rate (%) and analyzed using the chi-square or Fisher’s exact test, with statistical significance set at *P* < 0.05.

## Results

Comparison of general conditions between two groups of patients

This study included 876 fresh transfer cycles, with 296 using GnRH-ant and 580 using GnRH-a. The general conditions of patients are compared in Table 1. There were no significant differences between the two groups in any of the baseline characteristics including age, infertility duration, causes, BMI, AMH, and infertility duration (*P* > 0.05). The history of oocyte retrieval was higher(1.59 ± 0.90 versus 1.32 ± 0.69, *P *= 0.003) in the GnRH-ant group than in the GnRH-a group. History of pregnancies was more (1.00 ± 1.22, 0.69 ± 0.98, *P *= 0.045) in the GnRH-a group than in the GnRH-ant group, as shown in Table 1. 

**Table 1:** Comparison of baseline characteristics of participants in the two groups. Data are presented as number (*n*), mean ± SD for continuous variables, and percentage (%) for categorical variables. Statistical significance is set at *P* < 0.05. SD, standard deviation; AFC, antral follicle count; AMH, anti-Müllerian hormone; BMI, body mass index; GnRH-a, gonadotropin-releasing hormone agonist; GnRH-ant, gonadotropin-releasing hormone antagonist

Items	GnRH-a group	GnRH-ant group	*P*-value	*F*-value
Age of female (years)	30.21 ± 3.30	30.63 ± 3.45	0.325	0.461
Age of male (years)	31.42 ± 31.57	31.57 ± 4.60	0.782	0.001
BMI of female (kg/m^2^)	21.74 ± 1.88	22.11 ± 1.85	0.123	0.075
History of oocyte retrieval	1.32 ± 0.69	1.59 ± 0.90	0.003	12.543
History of pregnancies	1.00 ± 1.22	0.69 ± 0.98	0.045	1.645
History of labor	0.30 ± 0.53	0.26 ± 0.54	0.643	0.347
History of miscarriages	0.03 ± 0.19	0.01 ± 0.12	0.624	0.978
AFC	18.73 ± 8.43	19.12 ± 10.00	0.724	0.724
AMH (ng/mL)	7.55 ± 6.19	4.26 ± 3.86	0.753	0.295
Infertility duration (years)	3.67 ± 2.61	3.90 ± 3.07	0.646	0.608
Primary infertility (%)	46.38 (269/580)	53.62 (311/580)	0.510	0.434
Secondary infertility (%)	50.00 (48/96)	50.00 (48/96)	NA	NA

Comparison of baseline hormonal levels in the two groups of patients

Table 2 compares the medians of the baseline serum hormone levels (FSH, LH, E2, and P) in the two groups. It was found that there was no significant difference in the baseline hormonal levels (*P* > 0.05).

**Table 2:** Comparison of baseline hormonal levels of participants in the two groups of patients. Data are presented as number (*n*), mean ± SD. Clinical significance is set at *P* < 0.05. SD, standard deviation; bFSH, basal follicle-stimulating hormone; bLH, basal luteinizing hormone; bE2, basal estradiol; bP, basal progesterone; GnRH-a, gonadotropin-releasing hormone agonist; GnRH-ant, gonadotropin-releasing hormone antagonist

Items	GnRH-a group	GnRH-ant group	*P*-value	*F*-value
bFSH (Ｕ/L)	7.21 ± 3.68	132.68 ± 1001.33	0.142	34.6
bLH (Ｕ/L)	4.98 ± 5.38	6.15 ± 5.67	0.101	3.68
bE2 (pg/mL)	341.91 ± 980.86	459.63 ± 1273.31	0.378	2.862
bP (ng/mL)	1.66 ± 5.59	0.92 ± 2.12	0.302	2.472

Comparison of fertilization and embryo development in the two groups of patients

Table 3 compares fertilization and embryo development between the two patient groups. The GnRH-a group showed significantly higher values for total embryos transferred, day 2-3-4 embryos transferred, total cleavage counts, metaphase II (MII) numbers, total 2PN cleavage counts, total number of fertilizations, and percentage of high-quality embryo transfers compared to the GnRH-ant group (*P* < 0.05). There were no significant differences observed in endometrial thickness, day 5-6-7 embryos transferred, retrieved oocytes, total degradation number, and hCG day AFC between the two groups (*P* > 0.05).

**Table 3:** Comparison of fertilization and embryo development in the two groups of patients. Data are presented as number (*n*), mean ± SD for continuous variables. Clinical significance is set at *P* < 0.05. SD, standard deviation; PN, pronucleus; hCG, human chorionic gonadotropin; GnRH-a, gonadotropin-releasing hormone agonist; GnRH-ant, gonadotropin-releasing hormone antagonist

Items	GnRH-a group	GnRH-ant group	*P*-value	*F*-value
Total embryo transferred	0.95 ± 0.846	0.69 ± 0.872	0.016	1.65
Endometrium thickness	11.37 ± 2.42	10.95 ± 2.58	0.303	0.613
D3 embryo transferred	1.33 ± 0.88	0.92 ± 0.94	0.000	2.26
D5 embryo transferred	0.12 ± 0.44	0.11 ± 0.44	0.911	0.036
Retrieved oocytes	9.84 ± 5.92	8.28 ± 7.84	0.073	2.427
Total cleavage count	5.67 ± 4.28	3.67 ± 4.13	0.000	0.178
MII numbers	1.12 ± 1.43	0.75 ± 1.15	0.035	5.981
Total 2PN cleavage count	4.44 ± 3.54	2.98 ± 3.57	0.002	0.019
Total number of fertilization	5.89 ± 4.37	3.95 ± 4.25	0.001	0.018
Total degradation number	0.26 ± 0.74	0.26 ± 0.67	0.992	0.016
High-quality embryo transfer (%)	61.55 (357/580)	45.83 (44/96)	0.004	8.4331
HCG day AFC	10.72 ± 6.17	9.89 ± 5.08	0.210	2.728

Comparison of ovulation inductions in the two groups of patients

Significant differences were observed in the total dosage of Gn, the duration of Gn stimulation, intima thickness, LH, and E2 between the two groups. Total Gn (IU) dosages and duration of Gn stimulation were lower in the GnRH-ant group than in the GnRH-a group (*P* < 0.05). Intima thickness and serum LH and E2 levels were higher in the GnRH-ant group than in the GnRH-a group (*P* < 0.05). However, there were no significant differences in levels of FSH and P in the two groups (Table 4).

**Table 4:** Comparison of ovulation induction in the two groups of patients. Data are presented as number (*n*), mean ± SD. Statistical significance is set at *P* < 0.05. SD, standard deviation; FSH, follicle-stimulating hormone; LH, luteinizing hormone; E2, estradiol; P, progesterone; Gn, gonadotropin; GnRH-a, gonadotropin-releasing hormone agonist; GnRH-ant, gonadotropin-releasing hormone antagonist

Items	GnRH-a group	GnRH-ant group	*P*-value	*F*-value
Total dosage of Gn (IU)	2364.37 ± 917.12	1973.16 ± 966.22	0.001	0.638
Duration of Gn stimulation (day)	10.26 ± 3.05	8.75 ± 3.93	0.000	6.49
Endometrial thickness	3.02 ± 1.63	5.03 ± 2.54	0.000	30.182
Serum FSH (U/L)	5.12 ± 23.80	6.91 ± 4.30	0.566	0.001
Serum LH (U/L)	2.08 ± 8.64	4.49 ± 3.48	0.035	0.273
Serum E2 (pg/mL)	23.81 ± 19.38	36.30 ± 28.52	0.000	12.857
Serum P (ng/mL)	0.57 ± 1.79	0.48 ± 0.35	0.720	0.086

Comparison of pregnancy outcomes between two groups

The clinical pregnancy rate and implantation rate were higher in the GnRH-a group (61.54 versus 47.30, *P* = 0.037) compared to the GnRH-ant group (42.59 versus 26.01, *P *= 0.007), as shown in Table 5.

**Table 5:** Comparison of clinical outcomes in the two groups of patients. Data is presented as number (*n*), mean ± SD for continuous variables, and percentage (%) for categorical variables. Statistical significance is at *P* < 0.05. SD, standard deviation; OHSS, ovarian hyperstimulation syndrome; GnRH-a, GnRH agonist; GnRH-ant, GnRH antagonist; χ^2^, chi-square

Items	GnRH-a (*n* = 580)	GnRH-ant (*n* = 296)	*P*-value	*χ*^2^
Embryo transferred cycles (n)	351	148	NA	NA
Clinical pregnancy rate (%)	61.54 (216/351)	47.30 (70/148)	0.037	4.327
Live birth rate (%)	55.84 (196/351)	47.30 (70/148)	0.191	1.706
Implantation rate (%)	42.59 (247/580)	26.01(77/296)	0.007	7.146
Cancellation rate (%)				
Pre-ovulation	1.90 (11/580)	1.35 (4/296)	0.162	1.956
Egg retrieval	0.690 (4/580)	0.676 (2/296)	0.003	8.515
Embryo transfer	15.86 (92/580)	13.85 (28/296)	0.002	9.986
OHSS incident rate (%)	0.34 (2/580)	0.676 (2/296)	0.041	4.232

## Discussion

In assisted reproductive technology for IVF embryo transfer and ICSI, the current mainstream ovulation induction regimens use GnRH-ant and GnRH-a protocols, in which early follicular phase long-acting prolonged regimens are standard [16-17]. Many studies have analyzed and compared the clinical outcomes of the two regimens in different patient populations. Antagonists are increasingly widely used in clinical practice due to their short ovulation induction time and low Gn dosage. A meta-analysis compared the clinical outcomes of long GnRH-a regimens and GnRH-ant regimens in patients with a normal ovarian reserve and found that compared with GnRH-a regimens, GnRH-ant regimens significantly reduced the incidence of OHSS without affecting the pregnancy rate or live birth rate. Studies [18-21] have shown no significant differences in the number and quality of oocytes and embryos, the biochemical pregnancy rate, and the clinical pregnancy rate among the population of PCOS patients regarding the follicular phase, luteal phase, and antagonist regimens. It also showed that for PCOS patients and couples with poor response, the GnRH-ant regimen can reduce the occurrence of OHSS without reducing clinical pregnancy rates and can be considered the standard treatment for PCOS patients. A study [22] showed that in the population of poor ovarian response (POR) patients, the clinical outcomes of the long-acting agonist protocols in the early follicular phase are similar to those of the antagonist plan. However, age is an essential factor affecting the clinical pregnancy outcomes of IVF, and early pregnancy is the key to improving clinical outcomes. Therefore, the antagonist plan is preferred in the population of POR patients. These data indicate that the antagonist regimen is the preferred option in the population of ovarian hyperresponsiveness, PCOS patients, and POR patients.

Another study [23] also indicated that antagonists in the normal response population can achieve implant rates, pregnancy rates, and live birth rates similar to agonists and that the antagonist regimen is the preferred option. In the general population of IVF patients, although the GnRH-ant regimen can reduce the incidence of OHSS, its sustained pregnancy rate is lower than that of the long GnRH-a regimen. Studies [24-26] have shown that the GnRH-a regimen group showed a significantly higher endometrial thickness and pregnancy rate than the GnRH-ant regimen group, indicating higher endometrial receptivity. Due to its short ovarian stimulation period and low Gn dose, the GnRH antagonist treatment has been employed extensively in both high and poor ovarian responders [27-29]. In clinical practice, the ovarian response to ovulation induction is typically predicted using the following factors: patient age, baseline FSH, AFC, and AMH. Patients who are under 35 years of age and have a basal FSH level of less than 10 IU/L and an AFC level of more than five are considered normal ovarian responders [30-31]. Consequently, we used these parameters to choose normal ovarian responders for this study. We retrospectively examined the clinical results of the two distinct protocols, the GnRH-a and GnRH-ant protocols, to provide more references for clinical practice.

The data from this study shows that the number of eggs obtained and the blastocyst formation rate in the long-term agonist plan during the follicular phase are higher than those in the antagonist plan. The cancellation rate of fresh embryo transfer cycles is lower, and the clinical pregnancy rate is higher. Therefore, data from long-term GnRH-a studies in the population with normal ovarian reserves indicate that low cancellation rates of fresh cycle embryo transfers and higher clinical pregnancy rates during the follicular phase can effectively shorten the time required for patients to reach pregnancy.

This study’s limitation was its retrospective nature and the smaller sample size of patients in the GnRH-ant group. These factors may have led to imbalanced comparisons, potentially affecting the quality of evidence provided. Moreover, cases of frozen embryo transfers were not included.

## Conclusions

In this study, the fresh embryo implantation rate and clinical pregnancy rate of the GnRH-a regimen were higher than those of the GnRH-ant regimen. GnRH-a can effectively improve endometrial receptivity, facilitate embryo implantation, increase the cycle rate of fresh embryo transfers and clinical pregnancy rate, and reduce both the time required for patients to reach pregnancy and the economic costs for each sustained pregnancy. More data are required for a better comparative analysis.
